# Humpback whale “super-groups” – A novel low-latitude feeding behaviour of Southern Hemisphere humpback whales (*Megaptera novaeangliae*) in the Benguela Upwelling System

**DOI:** 10.1371/journal.pone.0172002

**Published:** 2017-03-01

**Authors:** Ken P. Findlay, S. Mduduzi Seakamela, Michael A. Meÿer, Stephen P. Kirkman, Jaco Barendse, David E. Cade, David Hurwitz, Amy S. Kennedy, Pieter G. H. Kotze, Steven A. McCue, Meredith Thornton, O. Alejandra Vargas-Fonseca, Christopher G. Wilke

**Affiliations:** 1 Mammal Research Institute Whale Unit, University of Pretoria, Pretoria, Wynberg, South Africa / Current address: Research Chair: Oceans Economy, Cape Peninsula University of Technology, Cape Town, South Africa; 2 Department of Environmental Affairs, Branch Oceans and Coasts, Victoria & Alfred Waterfront, Cape Town, South Africa; 3 Mammal Research Institute Whale Unit, University of Pretoria, Pretoria, 0001, South Africa [c/o 16 Ebor Rd, Wynberg, South Africa] / Current address: Marine Stewardship Council, 1 Snow Hill, London, United Kingdom; 4 Department of Biology, Hopkins Marine Station, Stanford University, 120 Ocean View Blvd, Pacific Grove, CA, United States of America; 5 12 Victory Way, Simon’s Town. Cape Town, South Africa; 6 The Marine Mammal Laboratory, Alaska Fisheries Science Center, 7600 Sand Point Way NE. Seattle, WA, United States of America; 7 Mammal Research Institute Whale Unit, University of Pretoria, Pretoria, Wynberg, South Africa; 8 ConserBio Foundation, Heredia, Costa Rica; 9 Department of Agriculture, Forestry and Fisheries, Branch: Fisheries Management, Foretrust Building, Foreshore, Rogge Bay, Cape Town, South Africa; Hampden Sydney College, UNITED STATES

## Abstract

Southern Hemisphere humpback whales (*Megaptera novaeangliae*) generally undertake annual migrations from polar summer feeding grounds to winter calving and nursery grounds in subtropical and tropical coastal waters. Evidence for such migrations arises from seasonality of historic whaling catches by latitude, *Discovery* and natural mark returns, and results of satellite tagging studies. Feeding is generally believed to be limited to the southern polar region, where Antarctic krill (*Euphausia superba*) has been identified as the primary prey item. Non-migrations and / or suspended migrations to the polar feeding grounds have previously been reported from a summer presence of whales in the Benguela System, where feeding on euphausiids (*E*. *lucens*), hyperiid amphipods (*Themisto gaudichaudii)*, mantis shrimp (*Pterygosquilla armata capensis*) and clupeid fish has been described. Three recent research cruises (in October/November 2011, October/November 2014 and October/November 2015) identified large tightly-spaced groups (20 to 200 individuals) of feeding humpback whales aggregated over at least a one-month period across a 220 nautical mile region of the southern Benguela System. Feeding behaviour was identified by lunges, strong milling and repetitive and consecutive diving behaviours, associated bird and seal feeding, defecations and the pungent “fishy” smell of whale blows. Although no dedicated prey sampling could be carried out within the tightly spaced feeding aggregations, observations of *E*. *lucens* in the region of groups and the full stomach contents of mantis shrimp from both a co-occurring predatory fish species (*Thyrsites atun*) and one entangled humpback whale mortality suggest these may be the primary prey items of at least some of the feeding aggregations. Reasons for this recent novel behaviour pattern remain speculative, but may relate to increasing summer humpback whale abundance in the region. These novel, predictable, inter-annual, low latitude feeding events provide considerable potential for further investigation of Southern Hemisphere humpback feeding behaviours in these relatively accessible low-latitude waters.

## Introduction

Southern Hemisphere humpback whales (*Megaptera novaeangliae*) are generally understood to migrate seasonally between summer high-latitude Antarctic feeding grounds and low-latitude winter calving and mating grounds in tropical and subtropical coastal waters of Southern Hemisphere continents and island archipelagos [1–4]. Evidence for such migrations arises from the timing and seasonality of historic whaling catches [5–12] and contemporary sighting survey results [13, 14] by latitude, *Discovery* mark ([2, 15, 16] and natural mark returns, [17, 18] as well as results of satellite tagging studies [19–25]. Of the seven Breeding Stocks recognised in the Southern Hemisphere by the International Whaling Commission [26], the migrations of the B Breeding Stock occurs between breeding grounds off the west coast of southern and central Africa (the coast between Angola and Gabon) and the Southern Ocean. Although historical catches were recorded off the Cape coast of South Africa and Namibia in the austral winter, spring and summer [6, 9, 12], it has been argued that the majority of the population migrates offshore along the Walvis Ridge to the west of the southern Benguela region [6, 9, 27]. At the northern limit of Southern Hemisphere humpback whale migrations, Rasmussen *et al*. [4] found a direct correlation between sea surface temperature and the location of wintering areas, indicative that warmer waters (of between 21.1 and 28.3°C) are integral in defining winter breeding migration patterns. The prevailing cold temperate oceanographic conditions in the Benguela Upwelling System off the west coast of Southern Africa results in the breeding grounds on this coast being significantly further north (0–16°S) than off the east coast (13–26°S).

Feeding has long been understood on the whole to be seasonally limited to summer and spatially limited within the southern high-latitude polar regions [3, 9, 10, 28, 29], with analyses of stomach contents of whales caught outside the Southern Ocean in the winter season often showing little or no food in their stomachs [3, 9, 30–32]. In Southern Ocean regions, Antarctic krill (*Euphausia superba*) has been identified as the primary prey item within stomachs examined on whaling flensing platforms [3, 9, 10, 33] and the distribution of humpback whales during the feeding season in Southern Ocean waters appears strongly coupled to that of these krill [34–36]. During the migrations to low latitude waters humpback whales are understood to survive off energy reserves accumulated during summer feeding as whales caught at the end of winter in the low latitude grounds were recorded to be considerably leaner with lower oil yields than those caught early in winter [31]. Whereas Corkeron and Connor [37] noted that the summer humpback whale migrations to the high latitudes are easily understood as feeding migrations [38–40], there is also considerable evidence that some populations, including those off the west coasts of South America [41] and southern Africa [42–44], suspend their southward migrations to polar waters whilst others may remain in productive temperate waters during the non-breeding season. Furthermore, a non-migratory humpback whale population in the Arabian Sea has been described by Minton *et al*. [45]. Records of feeding by humpback whales during their migrations in lower latitudes have been reported, although many of these records are of individual animals or small groups in apparent opportunistic feeding behaviours [46–53]. The more regular encounters of feeding from the Southern Benguela region [42–44], northern Patagonia [41] and the Magellan Straits of Chile [54] and the Eden coast of New South Wales, Australia, [55, 56] suggest that mid-latitude feeding and suspended migrations do occur on all three of these continents, although feeding is at a relatively low frequency compared to feeding at high latitudes.

The distributions of feeding baleen whales are good indicators of oceanographic productivity because these whales require dense and predictable prey aggregations for successful foraging [34, 57]. The productive coastal Benguela Upwelling System extends from between 14–17°S to 37°S off the southern African west coast. Distinct upwelling cells occur within the System, the most intense of which is the almost permanent Lüderitz upwelling cell (in the region of about 26°S to 27°S) that effectively divides the ecosystem into physically and biologically distinct northern and southern subsystems [58]. This study focussed within the southern Benguela subsystem between St Helena Bay and Cape Point, the oceanography of which is dominated by seasonal wind cycles of predominantly south-easterly offshore winds in summer and north-westerly winds in winter, with the summer south-easterly winds (October to March) resulting in pulsed, wind-driven coastal upwelling [58, 59]. The high productivity and abundant concentrations of zooplankton associated with the upwelling [60] results in the southern Benguela area being an important nursery ground for several fish species of ecological and commercial importance, including small planktivorous pelagic shoaling species such as sardine (*Sardinops sagax*) and anchovy (*Engraulis encrasicolus*) [58, 59].

Historic catches (1909 to 1928) made at the shore-based whaling stations in the Saldanha Bay region on the west coast of South Africa showed a bimodal [61] migration pattern believed to comprise the northward (July/August) and southward (October/ November) migrations [6, 8], with Olsen [6] noting that the whaling seasons from 1912 to 1913 were relatively long compared to other regions lasting till mid-December. However, Olsen [6] translated in Hinton [61] also reported that “In the cold currents of Saldanha Bay one could meet single young males probably yearlings, throughout the whole summer”, although the provenance of this information is questioned by Findlay and Best [62] as the whalers only operated between the months of March and late December. Best *et al*. [42] hypothesised that the suspension of the southward migration of humpback whales on the west coast of South Africa was in response to locally abundant prey. Historic reports of the presence of humpback whales within the northern Benguela subsystem during summer months arise from Townsend’s [63] records of 19^th^ century catches in January off Walvis Bay, Namibia (23°S) and a comment of Keeler’s (in [64]) that many humpback whales were found off Hollam’s Bird Island, Namibia in January 1829. In January, the majority of animals are expected to be on the Antarctic feeding grounds.

Notwithstanding the numerous reports of apparent or suspected feeding by humpback whales within the Benguela Upwelling System [6, 9, 42, 44, 62], or some evidence for relatively large loose feeding associations (*ca*. 20 individuals) at a local scale [46] this paper describes a unique humpback whale feeding behaviour that has been observed during the months of October and November in recent years (2011, 2014 and 2015) at a much wider regional scale within the southern Benguela sub-system.

## Methods and materials

In this paper, we use the term “super-group” to describe groups of 20 or more individual humpback whales estimated to be within five body lengths of their nearest neighbour. Such observations are novel in that prior observations of feeding by humpback whales in the region (e.g. [42–44]) were of loose aggregations of small groups of whales (of up to 20 individuals). These records of the “super-groups” of humpback whales within the southern Benguela region arise from two sources, namely primary observations from dedicated research cruises, and incidental observations reported by the general public.

**Fig 1:**
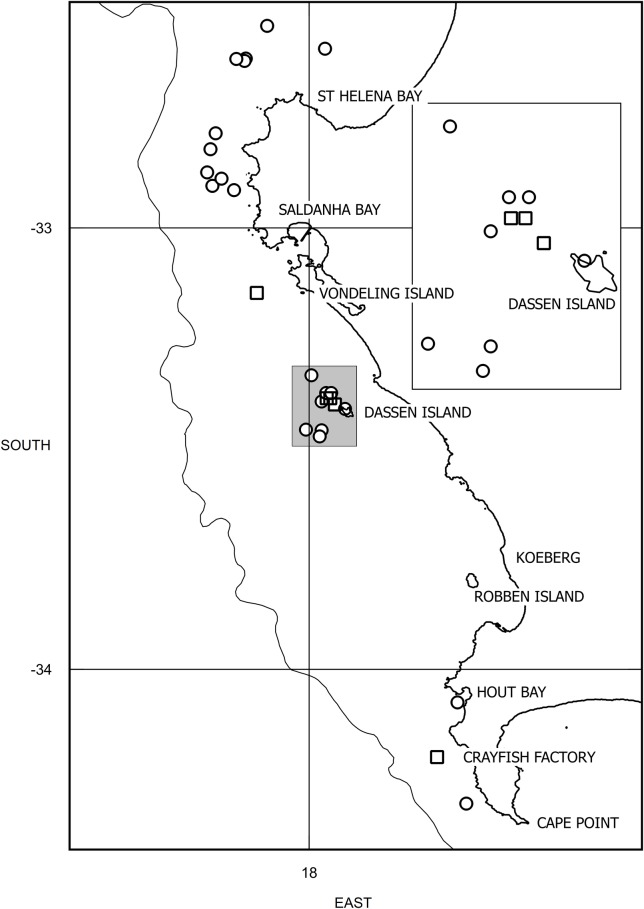
Locations of the Study Region and dedicated (open circles) and incidental observations (open squares) of feeding “super-groups” off the south west Cape coast of South Africa. The 200m isobath is shown. The inset expands the area shaded in grey.

Three dedicated research cruises were carried out in coastal waters between 32°20’S and 34°20’S off the south-western Cape region of South Africa (Fig 1) in late October–early November of 2011, 2014 and 2015 and focussed on identifying the distribution, relative abundance and movements of migratory large whales and other cetacean fauna in relation to biotic and abiotic factors (including upwelling areas) and the trophic ecology of large whales within this coastal area. The 2011 cruise was conducted from 10 November to 20 November, the 2014 cruise from 28 October to 8 November, while the 2015 cruise was conducted from 29 October to 7 November. The 2011 cruise was conducted aboard the South African Department of Environmental Affairs’ research vessel *RV Algoa*, while both of the 2014 and 2015 cruises were carried out aboard the South African Department of Agriculture Forestry and Fisheries research vessel the *FRS Ellen Khuzwayo* (hereafter both vessels are referred to as “mothership” below). During 2014 and 2015 concurrent oceanographic cruises (to those carried out on the *FRS Ellen Khuzwayo*) were carried out in the region aboard the *RV Algoa*. Due to limitations in the launching and recovery of rigid hull inflatable boats (RHIBs) from the *FRS Ellen Khuzwayo* in the prevailing south-westerly swell conditions during 2014 and 2015 this vessel generally anchored in sheltered conditions at night with the overnight position defining the area of search effort the following day. Searching for whales was generally initiated each morning from the bridge or monkey island of the mothership *en route* to the 100m isobath (some 5 n. miles offshore) and positions of whale groups were communicated by radio to one or two RHIBs following astern or parallel to the *FRS Ellen Khuzwayo* if conditions for RHIB operations allowed. Once approachable groups of whales were sighted, research personnel were transferred to the RHIBs for approach of whale groups, and RHIBs could be directed from whale group to whale group by the *FRS Ellen Khuzwayo*, until late afternoon when personnel were transferred back to the ship prior to seeking a sheltered anchorage or providing a lee, if swell conditions allowed for the recovery of the RHIBs. In 2011, the RHIBs were deployed from the *RV Algoa* at sea only once the first approachable whale group was sighted, and thereafter travelled astern or parallel to the mothership between whale groups. Where weather conditions precluded RHIB operations, whale groups were approached by the mothership. All approaches to whale groups and all research activities reported in this study were carried out under research permits (numbers RES2011/70, RES2014/61, RES2015/94) granted to Mammal Research Institute, University of Pretoria by the South African Department of Environmental Affairs and research permit (number RES2015/DEA) granted to the South African Department of Environmental Affairs by the Department of Agriculture, Forestry and Fisheries under provisions of the South African Marine Living Resources Act (Act 18 of 1998) and under ethics permits of the University of Pretoria, South Africa (Reference numbers—EC020-12 and EC061-15).

Due to the tight spacing and repeated diving behaviour of whales within “super-groups” (at times less than 2–3 m apart) and the high risk of entangling whales within sampling gear, no plankton sampling could be carried out from the RHIBs in the near vicinity of these groups. Group sizes of dedicated sightings were estimated independently by experienced observers as upper, lower and best estimates during the close approaches of the group, either by RHIBs or by the mothership so that group size estimates were not carried out at distance and consequently not influenced by Beaufort Scale or Sea State. Best estimates of group size were not necessarily the mean of the overall upper and lower estimates as independent estimates were made by all of the personnel observing the group and the final estimated group size was based on consensus of these individual estimates. The estimates of the sizes of “super-groups” pertain only to those individuals within five body lengths of the nearest neighbour at the time of the largest aggregation during the encounter. Fluidity of the associations within groups and movement of identified individuals between groups within aggregations (including in and out of “super-groups”) was difficult to determine in the field, due to the repetitive deep dive behaviour of the individual whales, which made the determination of group sizes difficult. Where such difficulty precluded accurate group size estimation, the lowest estimate was selected.

Incidental observations of “super-groups” were reported by the general public from aircraft during 2015 including during two sightseeing flights made by DH between Koeberg and St Helena Bay (including offshore of Dassen Island) on 19 October and 26 October 2015. These two flights were carried out at over 300m above sea level in a Cessna 185 aircraft. All of the reported incidental observations were verified from photographs. Group size estimates of the incidental sightings were estimated by the observers who recorded these sightings. Although other reports were received from boat-based and shore-based observers these have been excluded from the database as they were not accompanied by adequate photographic verification.

## Results

**Fig 2:**
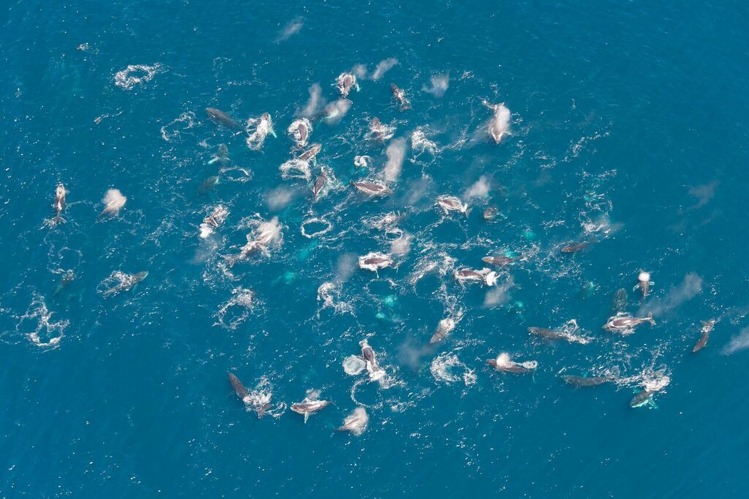
Aerial incidental observation (Observation 7, Table 2) of a “super-group” encountered some 5 km west of Crayfish Factory on the west coast of the Cape Peninsula, South Africa. Image courtesy of Jean Tresfon.

**Fig 3:**
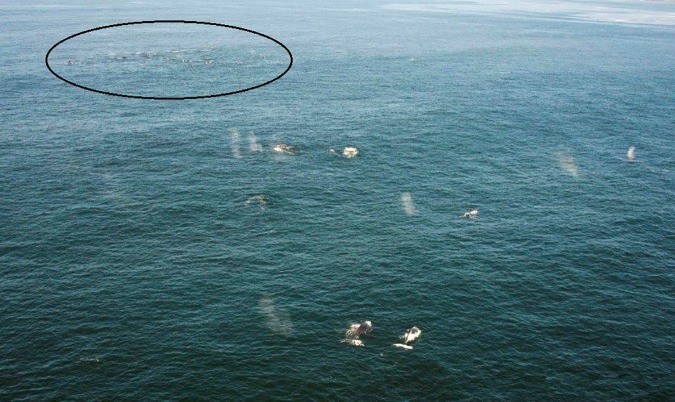
An aerial incidental observation of a “super-group” (Observation 3 Table 2, circled in background) within a widely distributed loosely-spaced aggregation of smaller feeding sub-groups (foreground) off Dassen Island.

“Super-groups” were defined as groups of 20 or more tightly-spaced individual humpback whales each estimated to be within five body lengths of their nearest neighbour (though in reality most “super-groups” were more tightly spaced than this) (Fig 2). In most cases our observations of “super-groups” occurred in the midst of more loosely-spaced aggregations of less dense, smaller sub-groups which were widely distributed over the observed range (Fig 3).

A total of 22 dedicated sightings of “super-groups” were made in the southern Benguela region during the three cruises (Table 1), whilst a further one was recorded during the oceanographic survey by the *R*.*V*. *Algoa* in 2015. At least seven incidental sightings were reported by the public including three sightings made from an aircraft on each of 16 October and 25 October 2015 and one sighting made from an aircraft on 4 November 2015 (Table 2). The locations of these primary sightings and the general area of the incidental sightings are shown in Fig 1. Water depths associated with the dedicated sightings ranged between 32 and 86 m. The sightings of these “super-groups” all arise from a localised coastal region some 200 nautical miles in extent within the highly productive southern Benguela Upwelling System and ranged from the Columbine upwelling cell in the north to the Cape Peninsula upwelling cell in the south. Positions of the “super-groups” did not appear to be related to any bathymetric features or depth intervals.

**Table 1:** Dedicated observations of “super-groups” encountered on cruises in the southern Benguela in 2011, 2014 and 2015.

Observation Number	Vessel	Date	Group size high	Group size low	Group size best	Latitude (S)	Longitude (E)	Comment
1	RV Algoa	12 November 2011	38	22	30	-32.6163	17.8566	
2	RV Algoa	12 November 2011	22	20	20	-32.62166	17.8538	
3	RV Algoa	14 November 2011	20	10	20	-32.5940	18.03605	
4	FRS Ellen Khuzwayo	28 October 2014	70	50	60	-33.3933	18.02833	
5	FRS Ellen Khuzwayo	29 October 2014	80	60	70	-33.4585	18.02833	
6	FRS Ellen Khuzwayo	29 October 2014	200	150	175	-33.457	17.99283	
7	FRS Ellen Khuzwayo	01 November 2014	*	*	60	-33.4724	18.02393	
8	FRS Ellen Khuzwayo	01 November 2014	*	*	80	-33.3339	18.00527	
9	FRS Ellen Khuzwayo	02 November 2014	*	*	20	-33.374	18.03868	Fin whale within group
10	FRS Ellen Khuzwayo	02 November 2014	25	20	20	-33.374	18.05	Fin whale within group
11	FRS Ellen Khuzwayo	05 November 2014	35	25	30	-32.6172	17.83518	
12	FRS Ellen Khuzwayo	05 November 2014	50	30	35	-32.5422	17.905	
13	FRS Ellen Khuzwayo	05 November 2014	*	*	30	-32.9047	17.781	Southern right whale within group
14	FRS Ellen Khuzwayo	05 November 2014	*	*	20	-32.9047	17.781	
15	FRS Ellen Khuzwayo	06 November 2014	*	*	70	-32.8884	17.8016	
16	FRS Ellen Khuzwayo	06 November 2014	*	*	80	-32.8884	17.8016	
17	FRS Ellen Khuzwayo	07 November 2014	30	20	25	-32.8217	17.77667	
18	FRS Ellen Khuzwayo	29 October 2015	60	40	40	-33.4100	18.08138	
19	FRS Ellen Khuzwayo	30 October 2015	150	100	120	-32.7856	17.78837	Two Southern right whales within group
20	FRS Ellen Khuzwayo	31 October 2015	60	40	50	-32.9144	17.83	
21	FRS Ellen Khuzwayo	05 November 2015	180	150	150	-34.3043	18.356	
22	FRS Ellen Khuzwayo	06 November 2015	30	20	20+	-34.075	18.33604	
23	RV Algoa	31 October 2015	35	25	30	-32.8742	17.76917	

*—Not Recorded.

**Table 2:** Incidental observations of “super-groups” made by the public in the southern Benguela in 2011, 2014 and 2015. All of these observations were made at altitudes exceeding 300m.

Observation	Observer	Date	Platform	Group size	Locality
1	David Hurwitz	19 October 2015	Aerial	50	5 n. miles west of Vondeling Island
2	David Hurwitz	19 October 2015	Aerial	100	2 n. miles NNW of Dassen Island
3	David Hurwitz	19 October 2015	Aerial	50	1 n. mile west of above observation
4	David Hurwitz	26 October 2015	Aerial	50	Just N-NW of Dassen Island
5	David Hurwitz	26 October 2015	Aerial	50	Just N-NW of Dassen Island
6	David Hurwitz	26 October 2015	Aerial	50	Just N-NW of Dassen Island
7	Jean Tresfon	4 November 2015	Aerial	60	5 km west of Crayfish Factory on the west coast of the Cape Peninsula

Best estimates of the sizes of the dedicated cruise sightings of “super-groups” of humpback whales ranged between 20 and 200 individuals (Table 1), although there was often considerable range in these estimates made by different observers, reflecting the difficulty of group size estimation of such large groups. Despite a minimum independent estimate of group size being the number of photo-identified individuals encountered in the group, such an estimate doesn’t take the high group flux into consideration. For example, at least 67 individuals were identified from tail fluke colouration patterns within the “super-group” observation of 28 October 2014, at which a best group size of 60 individuals was estimated at the time of sighting. Estimates of group sizes made by incidental observers ranged between 50 and 60 whales per group, all of which were carried out from aircraft at elevated observations and which may consequently have greater veracity. On the whole the “super-groups” were often located within large loose aggregations of humpback whales spread over the entire visual area from the mothership (estimated as an extended area of between 10 and 20 square kilometres). Furthermore, the flux of animals into and out of “super-groups” appeared high, with single animals, pairs and even subgroups of up to five individuals observed to join and leave “super-groups” during observations; some of these were observed to travel in towards the “super-group” from considerable distances.

At least 40 individuals are discernible in Fig 2 (image of incidental observation number 7 (Table 2) estimated by the observer as 60 individuals). The proportion of diving whales to whales observable at or near the surface is unknown, but given the observed rapid and repeated diving behaviour within these groups, is believed to be greater than parity, so that a group size estimate of 60 individuals is considered a probable underestimate.

**Fig 4:**
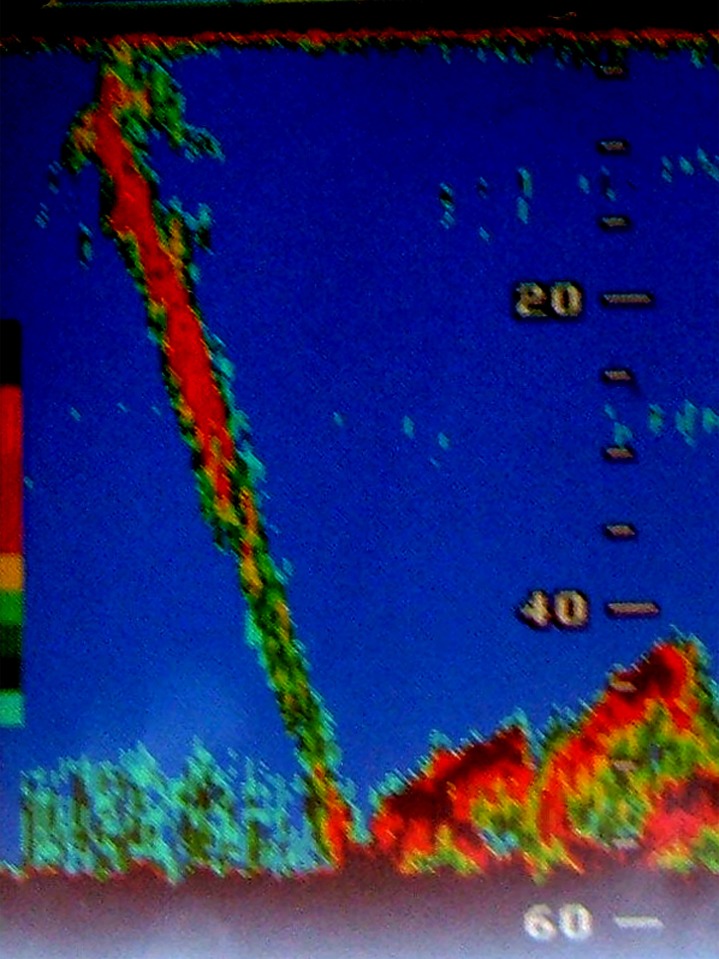
Photograph of a RHIB sonar echogram showing the trail of a diving whale to the seafloor within the dedicated observation of three whales on the edge of a “super-group” at 32.57°S; 18.048°E on 14 November 2011 (Observation 3 in Table 1).

Generally, the behaviour of animals within all “super-groups” appeared to be focussed on feeding, with immediate feeding being identified by observations of surface gaping and lunging and tight turning and repeated strong vertical diving behaviour whilst extended feeding behaviour was apparent from observations of defecations (including brick red solid faeces) and a pungent (“fishy”) odour of the whale blows, as opposed to the more normal oily odour of blows found on breeding grounds (KF pers. obs.). The repeated strong vertical diving behaviour predominated in these encounters, especially in 2014. Underwater exhalations and the formation of bubble clouds by individual whales were noted in a number of the “super-groups” and are clearly evident in Fig 2. Bird (Cape gannet (*Morus capensis*) and tern species) and Cape fur seal (*Arctocephalus pusillus pusillus*) feeding were associated with some super-groups. On two occasions a single fin whale (*Balaenoptera physalus*) was associated with super-groups, and on two occasions (06 November 2014 and 30 October 2015), southern right whales (*Eubalaena australis*) were present within a super-group. The RHIB echo-sounders often noted prey aggregations either at the bottom, mid-water or at the surface within the water column in the vicinity of “super-groups” although no verification of these targets could be determined through plankton tows due to the tight spacing of the whales. Whilst surface lunging was observed on occasion, whale behaviour within the “super-groups” often included a high incidence of repetitive fluke-up dives, indicative of feeding at some depth. On certain occasions whales could be tracked by the RHIB echo-sounder in the vicinity of such prey aggregations at the seafloor (Fig 4). Both mantis shrimp (*Pterygosquilla armata capensis*) and euphausiids (*Euphausia lucens*) were observed on the surface within super groups.

Whilst “super-groups” as a whole moved during observations, the direction of whale movements within the group was not necessarily tied to the direction of the group movement, although in some cases (particularly in the larger super-groups) the individual’s movement on the surface was in the general direction of the group movement. In most cases there was independent turning and milling of individuals within the super-groups, although synchronicity in diving and feeding was sometimes evident within sub-groups of up to five whales within the super-groups. “Super-groups” were observed to form rapidly with similar rapid dissociation before forming in new areas reasonably close to the previous area, presumably dependent on the concentrations of food resources in the water column. The distribution of “super-group” aggregations in 2015 appeared to shift southward over the period 19 October to 12 November with no groups being seen in the south at the start and no groups being seen in the north at the end of this period respectively.

Whilst large mature humpback whales were observed in super-groups, many of the observed individuals were small estimated at between 8 and 10 metres. Only one calf was sighted in the over 30 observations of “super-groups” (on 5 November 2015).

## Discussion

Southern Hemisphere humpback whales are generally understood to feed at the summer polar termini of their annual migrations, with the majority of evidence of such feeding arising from investigations of stomach contents of whales examined on flensing platforms (both in Antarctic region and lower latitude migration corridors and breeding grounds) [3, 9]. Although evidence for feeding during mid and low latitude migrations have been reported [41, 42, 44, 56, 62, 65], the predictable seasonal observations of novel feeding behaviour by such large groups of feeding humpback whales across at least five years (2011 to 2015) reported here are the first confirmed records of such large aggregation behaviour patterns within low latitude waters, and possibly surpass the sizes of feeding groups in both northern and southern polar waters. Neither Best *et al*. [42] nor Barendse *et al*. [44] report of group feeding behaviours in their shore-based observations and associated small boat surveys within this region in 1995 and between 2001 and 2003 respectively. One group of 20 whales was recorded by Barendse *et al*. [44], although the authors noted that this “in reality was a dynamic aggregation of several smaller groups” and a “Large, loosely associated group identified as 11 smaller groups from Land”: Such observations may have been precursors of the contemporary super-groups, but given the limited spatial extent of the shore-based studies and more-limited sighting conditions from shore, other larger groups could have remained undetected. Despite feeding humpback whales having been recorded by a number of authors within the southern Benguela region [6, 9, 42, 44], we propose that the “super-group” feeding phenomenon (as tightly spaced large groups of whales) is a relatively recent behaviour exhibited by these whales. Whilst the difficulty of the group size estimation is acknowledged, the estimates were made in close proximity to the groups so that the estimates are influenced only through the whale behavioural patterns rather than environmental variables. Furthermore, although aerial platforms may provide some elevated perspective, they do not increase the confidence of group size estimation as repeated surfacing bouts are impossible to link to particular individuals.

The novelty of the encountered behaviours is twofold, namely a) the formation of tightly-spaced large groups of humpback whales feeding intensely within concentrated areas (an intensity which appears to exceed that observed by two of the authors (MAM and KPF) in Antarctic waters), and b) the predictability of these feeding behaviours of humpback whales within low latitude waters. Nowacek *et al*’s. [36] description of high densities of humpback whales feeding on Antarctic krill in the Western Antarctic Peninsula report whale densities of 5.1/km^2^, an order of magnitude less than the group aggregations reported here. Furthermore, no such dense feeding aggregations have been reported elsewhere in low or mid latitudes during Southern Hemisphere humpback whale migrations. Indeed, aggregations of whales of this size have seldom been reported in the literature, with “large” groups often numbering in the range of 10–20 or less [66–68].

Barendse *et al*. [69] found that the study area is used during spring and summer by a small component of about 500 humpback whales which they thought were likely to migrate and breed within the tropical waters off Gabon in Central Africa. However, Carvalho *et al*. [70] noted that some of the whales feeding in the southern Benguela may be breeding and calving in an area that is yet to be identified. Whilst no abundance estimates have yet been generated from the photo-identification in the 2011, 2014 and 2015 surveys, the numbers of whales seen in both large “super-groups” and aggregations during the late October and early November 2014 and 2015 surveys must suggest a brief temporary immigration into the region that is well in excess of the 500 individuals estimated to occur in the region in summer by Barendse *et al*. [69]. Preliminary results of satellite tracking carried out in 2014 showing that six of eight individuals tagged within “super-groups” migrated southwards to the Southern Ocean by early summer (November), although four of these individuals first moved north for some 100 to 150 n. miles before migrating south [71]. However, whereas there is little current information on the migratory origin of the whales within the “super-groups”, there is some evidence to suggest that they may not necessarily be a suspended southward migration of whales from the breeding grounds off West Africa as proposed by Best *et al*. [42]. The southward migration route of humpback whales from the West Africa breeding grounds is likely further to the west of the study area, as appears to be the case for the northwards migration of these animals based on historical reports [6, 8, 61] and more recent scientific observations [44] showing little evidence for northwards migrating animals in the vicinity of Saldanha Bay. Furthermore, the migration tracks of two humpback whales satellite-tagged in the breeding grounds off Gabon as these animals travelled south-westwards along the Walvis Ridge were well to the west of our study domain during their southward migration [27]. The above and the combination of (a) the lack of encountered calves that would be expected at this time of year if the whales in “super-groups” were emanating from the breeding grounds and although Barendse et al. [69] and Findlay and Best [62] report of observations of single calves from the region at this time of year, the expected numbers should be far higher based on observations of calves on the east coast of South Africa during the southward migration [72] or during southward migrations off other continents [73]; (b) that whale disentanglement efforts in the region have shown links through gear-type to the south coast of the country during the previous months (MAM pers. obs.); and (c) the high incidence of yellow diatom films (presumed to be *Benetella ceticola*) on the skin of the whales found in this area is suggestive of a recent movement of whales from a cold water habitat (see [74]), and not from the tropics. We believe the animals encountered within “super groups” did not migrate further north than South African waters during the preceding austral winters and possibly comprises a non-breeding migration of young animals from Antarctic waters and the immigration of young non-breeding animals from Breeding Stock C during their southern migration. It should be noted that Dawbin [2] found that younger humpback whales could be caught on mid-latitude grounds during the mid-winter season when mature whales were on the more northerly breeding grounds. Furthermore, Olsen [6] reported that young male humpback whales were encountered in the southern Benguela during the summer months.

The identification of the prey species eliciting “super-group” feeding behaviours remains undetermined. Humpback whales have been recorded feeding on a number of prey species within the region. Although Barendse *et al*. [44] noted that the 26 observations of feeding behaviour and “apparent feeding behaviour” (including defecations) during spring and summer in the southern Benguela upwelling region between 2001 and 2003 provide evidence that the region may function as an important feeding area for these whales, they note that little evidence of prey species could be obtained, apart from possible euphausiid exoskeleton remains and a hyperiid amphipod found within collected faecal samples. Humpback whales have been recorded feeding in summer on copepods (“rodaate” in Norwegian from Olsen (1914) [6] translated in [44] and “herrings” [6], mantis shrimp (*P*. *armata capensis*) [62], euphausiids (*E*. *lucens*) and amphipods [42, 44] including hyperiid amphipod (*Themisto gaudichaudii)* [44] in the southern Benguela, while Matthews [9] found that of a total of eight stomachs of humpback whales examined at Saldanha Bay during winter, fish (“? Clupeiods” and “a pasty mash of fish scales and bones”) were present in two; one in June, 1926 and one in September, 1926. Best *et al*. [42] recorded apparent feeding behaviour by humpback whales on 10 occasions over their 38 days of shore-based observations and the observation of production of faeces indicative of recent feeding on 7 occasions during small boat approaches. Their collected faecal samples contained euphausiid remains (“possibly *E*. *lucens*”) on 2 occasions and amphipods on another. The available evidence points to considerable opportunistic feeding behaviour on a wide range of prey species from the region. Clear differences in the acoustic scattering of prey patches (measured at 200 kHz and 38 kHz from the *FRS Ellen Khuzwayo*) were highly suggestive of dense aggregations of euphausiids in 2015. In all the study years, the RHIB echo-sounders identified dense prey aggregations almost on the sea floor under the “super-groups” although the possibility of these aggregations being prey species other than *E*. *lucens* cannot be ruled out. During the 2014 observations of the super-groups, a co-occurring predatory fish species, Snoek (*Thyrsites atun*), were caught at Dassen Island with full stomach contents of stomatopod mantis shrimps (*P*. *armata capensis)* (CW pers. obs.). Although no such catches were made in 2015, mantis shrimps were observed in association with humpback whales on 06 November 2015 off Hout Bay and a mass stranding of mantis shrimps occurred in this bay within 14 days after the cruise. Furthermore, the only prey recorded in the stomach of a 6.4m humpback whale entangled in rock lobster fishing gear in 1990 (see [62]) was young adults of this stomatopod species which Griffiths and Blaine [75] found to be the only stomatopod species occurring to the west of Cape Point, with the distribution off the west coast corresponding to terrigenous mud beds. Although predominantly a benthic species, the occurrence of *P*. *armata capensis* in stomachs of other pelagic predators such as African penguins, cormorants and snoek, [75] suggests that pelagic swarming behaviour makes the species available to predators including baleen whales [62].

The recent observations of this novel and intense feeding behaviour is of particular interest in light of rapidly recovering humpback populations on both the east and west coasts of southern Africa and in the associated Southern Ocean region [14, 43, 76, 77] with the observation of this behaviour potentially arising from the following potential four scenarios:

1. alterations in prey availability leading to a novel feeding strategy;
2. increasing humpback whale abundance intensifying pressure on prey availability elsewhere and a consequential switch in feeding strategies or areas;
3. a restoration of a previously unobserved feeding strategy as the population abundances re-establish; or
4. an increase in the probability of detection of “super-group” behaviour as the population abundance increases.

Although Verheye *et al*. [78] describe long-term changes in neritic zooplankton communities in the southern Benguela subsystems with a shift in domination from large to smaller species, we currently remain uncertain as to the mechanism driving this novel low-latitude behaviour in Southern Hemisphere humpback whales. Given that Olsen [6] reported of a summer incidence of juvenile humpback whales in the region prior to 1914 (albeit not at such densities) the observed behaviour may well be a restoration of a previously unobserved feeding strategy as the population abundances re-establish. Furthermore, as noted by Owen *et al*. [55] the extent of feeding on migration could influence the extent to which humpback whales rely on the Antarctic ecosystem each year. Despite the unknown cause of this recent behaviour, we postulate that the area has developed / is developing into an important seasonal humpback whale feeding ground that attracts significant immigration into the region in the late austral spring / early summer. Whilst humpback whales in the region are clearly feeding opportunistically on a range of prey species, the concentrations, identity and size of the prey eliciting “super-group” feeding behaviours remain unknown and require investigation as we believe this will be a primary step in identifying the system changes (including whale population increases) that have resulted in these observations. Owen *et al*. [55] suggest that prey type may be important in influencing the extent of feeding on migration. Further required investigations include a) the origin of this immigration into the region as the lack of calves in the “super-group” encounters and the high incidence of cold water diatoms on the whales is unexpected at this time of year, b) observations of tagged or marked individuals within “super-groups” to obtain some information on the surfacing time to dive time ratios (along with aerial observations) for the modelling of group size estimation.

This novel, predictable (at least over the 2014–2015 period) and accessible feeding behaviour within low latitudes provides considerable opportunity for further investigation of Southern Hemisphere humpback feeding aggregations and behaviours outside of the relatively inaccessible ice-edge region of the Southern Ocean. Future areas of investigation should include identifying migration links and the population identity of participating whales, multiscale examination of the feeding ecology of these humpback “super-groups” (e.g. [79]) and modelling of the energetic advantages of the migration suspension.

## Supporting information

S1 Dataset Dedicated and incidental sightings of “super-groups” of humpback whales reported in this study. (DOCX)
