# Supplementary material for: Humpback whale “super-groups” – A novel low-latitude feeding behaviour of Southern Hemisphere humpback whales (Megaptera novaeangliae) in the Benguela Upwelling System
Source: PLoS One. 2017 Mar 1;12(3):e0172002. doi: 10.1371/journal.pone.0172002 (PMC5332018; doi:10.1371/journal.pone.0172002)
Supplement: S1 Dataset — (DOCX) [file pone.0172002.s001.docx]

1. Dedicated observations of “super-groups” encountered on cruises in the southern Benguela in 2011, 2014 and 2015.

| **Observation Number** | **Vessel** | **Date** | **Group size high** | **Group size low** | **Group size best** | **Latitude (S)** | **Longitude (E)** | **Comment** |
| --- | --- | --- | --- | --- | --- | --- | --- | --- |
| 1 | *RV Algoa* | 12 November 2011 | 38 | 22 | 30 | -32.6163 | 17.8566 |  |
| 2 | *RV Algoa* | 12 November 2011 | 22 | 20 | 20 | -32.62166 | 17.8538 |  |
| 3 | *RV Algoa* | 14 November 2011 | 20 | 10 | 20 | -32.5940 | 18.03605 |  |
| 4 | *FRS Ellen Khuzwayo* | 28 October 2014 | 70 | 50 | 60 | -33.3933 | 18.02833 |  |
| 5 | *FRS Ellen Khuzwayo* | 29 October 2014 | 80 | 60 | 70 | -33.4585 | 18.02833 |  |
| 6 | *FRS Ellen Khuzwayo* | 29 October 2014 | 200 | 150 | 175 | -33.457 | 17.99283 |  |
| 7 | *FRS Ellen Khuzwayo* | 01 November 2014 | * | * | 60 | -33.4724 | 18.02393 |  |
| 8 | *FRS Ellen Khuzwayo* | 01 November 2014 | * | * | 80 | -33.3339 | 18.00527 |  |
| 9 | *FRS Ellen Khuzwayo* | 02 November 2014 | * | * | 20 | -33.374 | 18.03868 | Fin whale within group |
| 10 | *FRS Ellen Khuzwayo* | 02 November 2014 | 25 | 20 | 20 | -33.374 | 18.05 | Fin whale within group |
| 11 | *FRS Ellen Khuzwayo* | 05 November 2014 | 35 | 25 | 30 | -32.6172 | 17.83518 |  |
| 12 | *FRS Ellen Khuzwayo* | 05 November 2014 | 50 | 30 | 35 | -32.5422 | 17.905 |  |
| 13 | *FRS Ellen Khuzwayo* | 05 November 2014 | * | * | 30 | -32.9047 | 17.781 | Southern right whale within group |
| 14 | *FRS Ellen Khuzwayo* | 05 November 2014 | * | * | 20 | -32.9047 | 17.781 |  |
| 15 | *FRS Ellen Khuzwayo* | 06 November 2014 | * | * | 70 | -32.8884 | 17.8016 |  |
| 16 | *FRS Ellen Khuzwayo* | 06 November 2014 | * | * | 80 | -32.8884 | 17.8016 |  |
| 17 | *FRS Ellen Khuzwayo* | 07 November 2014 | 30 | 20 | 25 | -32.8217 | 17.77667 |  |
| 18 | *FRS Ellen Khuzwayo* | 29 October 2015 | 60 | 40 | 40 | -33.4100 | 18.08138 |  |
| 19 | *FRS Ellen Khuzwayo* | 30 October 2015 | 150 | 100 | 120 | -32.7856 | 17.78837 | Two Southern right whales within group |
| 20 | *FRS Ellen Khuzwayo* | 31 October 2015 | 60 | 40 | 50 | -32.9144 | 17.83 |  |
| 21 | *FRS Ellen Khuzwayo* | 05 November 2015 | 180 | 150 | 150 | -34.3043 | 18.356 |  |
| 22 | *FRS Ellen Khuzwayo* | 06 November 2015 | 30 | 20 | 20+ | -34.075 | 18.33604 |  |
| 23 | *RV Algoa* | 31 October 2015 | 35 | 25 | 30 | -32.8742 | 17.76917 |  |
|  |  |  |  |  |  |  |  |  |

* - Not Recorded

1. Incidental observations of “super-groups” made by the public in the southern Benguela in 2011, 2014 and 2015. All of these observations were made at altitudes exceeding 300m.

| Observation | Observer | Date | Platform | Group size | Locality |
| --- | --- | --- | --- | --- | --- |
| 1 | David Hurwitz | 19 October 2015 | Aerial | 50 | 5 n. miles west of Vondeling Island |
| 2 | David Hurwitz | 19 October 2015 | Aerial | 100 | 2 n. miles NNW of Dassen Island |
| 3 | David Hurwitz | 19 October 2015 | Aerial | 50 | 1 n. mile west of above observation |
| 4 | David Hurwitz | 26 October 2015 | Aerial | 50 | Just N-NW of Dassen Island |
| 5 | David Hurwitz | 26 October 2015 | Aerial | 50 | Just N-NW of Dassen Island |
| 6 | David Hurwitz | 26 October 2015 | Aerial | 50 | Just N-NW of Dassen Island |
| 7 | Jean Tresfon | 4 November 2015 | Aerial | 60 | 5 km west of Crayfish Factory on the west coast of the Cape Peninsula |
|  |  |  |  |  |  |
